# Etiology of End-Stage Renal Disease and Arterial Stiffness among Hemodialysis Patients

**DOI:** 10.1155/2017/2543262

**Published:** 2017-02-19

**Authors:** Balsam El Ghoul, Yazan Daaboul, Serge Korjian, Andrew El Alam, Anthony Mansour, Essa Hariri, Salam Samad, Pascale Salameh, Georges Dahdah, Jacques Blacher, Michel E. Safar, Sola Aoun Bahous

**Affiliations:** ^1^North Hospital Center, Zgharta, Northern Lebanon, Lebanon; ^2^Department of Medicine, Tufts Medical Center, Tufts University School of Medicine, Boston, MA, USA; ^3^Department of Medicine, Beth Israel Deaconess Medical Center, Harvard Medical School, Boston, MA, USA; ^4^Lebanese American University School of Medicine, Byblos, Lebanon; ^5^Lebanese University, School of Public Health, Beirut, Lebanon; ^6^Diagnosis Center, Hotel-Dieu Hospital, Paris, France; ^7^University Medical Center-Rizk Hospital, Beirut, Lebanon

## Abstract

*Background*. Prior studies have demonstrated that conventional and emerging CV risk factors are associated with worsening arterial stiffness among end-stage renal disease (ESRD) patients on hemodialysis. The present cross-sectional study evaluates the association between the etiology of ESRD and arterial stiffness among a cohort of hemodialysis patients.* Methods*. Etiology of ESRD was identified from patients' medical records and classified as either vascular renal disease, diabetic nephropathy, nondiabetic glomerulopathy, tubular interstitial nephropathy, hereditary nephropathy, or ESRD of unconfirmed etiology.* Results*. A total of 82 subjects were enrolled. cfPWV was independently associated with the composite of either diabetic nephropathy or vascular renal disease (*p* = 0.022), pulse pressure (*p* = 0.001), and a history of CV events (*p* = 0.025), but not history of hypertension or diabetes mellitus alone. The median cfPWVs in diabetic nephropathy and vascular renal disease were comparable and significantly higher than median cfPWVs in other etiologies of ESRD.* Conclusion*. The study suggests that the etiology of ESRD is independently associated with arterial stiffness among hemodialysis patients. Furthermore, arterial stiffness was higher among patients who developed renal sequelae of either diabetes mellitus or hypertension as compared with those who have a history of either diabetes mellitus or hypertension alone.

## 1. Introduction

Cardiovascular (CV) disease is the most common cause of morbidity and mortality among patients with end-stage renal disease (ESRD) on hemodialysis (HD) [1]. Compared with the general population, the incidence of CV events among these patients is significantly higher, but it does not seem to be fully explained by the increased incidence of conventional risk factors alone. It has been hypothesized that HD patients are exposed to unique renal- and HD-related risk factors that predispose them to an increased rate of CV events [2, 3].

Arterial stiffness, a functional marker of arterial disease, may be measured by carotid-femoral pulse wave velocity (cfPWV), a noninvasive and reproducible technique to evaluate large artery stiffness [4]. The independent association between cfPWV and CV risk has been previously demonstrated in several patient populations, including those on HD [2, 5]. Compared with age-matched controls, HD patients are at an increased risk of developing arterial stiffness and vascular calcifications, both of which may contribute to the development of CV disease and subsequent CV events [3, 4, 6]. While the physiological interaction between the kidney and the vascular system has been well established, the etiology of ESRD among HD patients has been poorly investigated in the context of arterial stiffness and CV risk. The present cross-sectional study evaluates the association between the etiology of ESRD and cfPWV in a cohort of HD patients.

## 2. Materials and Methods

### 2.1. Study Participants

A total of 93 subjects with ESRD undergoing HD at the North Hospital Center in Zgharta, Lebanon, and who had been stable for at least 3 months were invited to participate in this cross-sectional study. Of those, 82 subjects consented and were enrolled. All procedures performed were in accordance with the ethical standards of the institutional and/or national research committee and with the 1964 Declaration of Helsinki and its later amendments. All subjects provided written informed consent, and the study was approved by the North Hospital Center institutional review board.

### 2.2. Measurements

Relevant medical and biological patient information was obtained from patients' electronic medical records and confirmed by means of a personal interview, which included demographic information as well as confirmation of personal and family medical and social history and adherence with home medications. Etiology of ESRD was classified as either vascular renal disease, diabetic nephropathy, nondiabetic glomerulopathy, tubulointerstitial nephropathy, hereditary nephropathy, or ESRD of unconfirmed etiology. Vascular disease was defined as either nephroangiosclerosis (defined as either biopsy-proven or presumed based on either the presence of long-standing hypertension of nonrenal origin for >10 years, development of hypertensive target organ damage, or normal baseline glomerular filtration rate with progressive worsening of renal function) or ESRD due to renal artery stenosis. Diabetic nephropathy was defined as either severely increased albuminuria that is macroalbuminuria (urinary albumin excretion rate > 300 mg/24 hr) among subjects with prior history of diabetes mellitus or moderately increased albuminuria that is microalbuminuria (urinary albumin excretion rate between 30 and 300 mg/24 hr) among subjects with diabetic retinopathy or type I diabetes mellitus for more than 10 years [7]. ESRD secondary to diabetic nephropathy was defined as ESRD with a history of diabetic nephropathy and no evidence of other renal or systemic diseases. Nondiabetic glomerulopathy was biopsy-proven and was defined as glomerulopathy with no evidence of diabetes mellitus or features of diabetic nephropathy on biopsy. Tubulointerstitial nephropathy was defined as ESRD secondary to either pyelonephritis, analgesic nephropathy, congenital obstructive or nonobstructive malformations, such as reflux nephropathy, renal dysplasia, or acquired obstructive uropathy. Hereditary nephropathy was defined as either ESRD secondary to polycystic kidney disease, ESRD secondary to confirmed genetic etiology, or early-onset (<40 years) ESRD of undetermined etiology among patients with first-degree family history of early-onset ESRD.

Hypertension was defined as either intake of antihypertensive therapy or an average systolic blood pressure ≥ 140 mmHg and/or diastolic blood pressure ≥90 mmHg over 6 consecutive measurements. Diabetes mellitus was defined as either HbA1c > 6.4% or intake of antidiabetic drugs. History of CV events was defined as either prior acute coronary syndrome or stroke.

Aortic stiffness was estimated by the measurement of cfPWV using an automated device (Complior, France) as previously described and validated in the hemodialysis population [8]. All measurements were obtained one hour following a hemodialysis session by the same trained operators. Simultaneously recorded pulse waveforms were obtained transcutaneously over the common carotid and femoral arteries. cfPWV was then calculated automatically by the device using the distance between the carotid and femoral artery recording sites divided by the time interval of the pressure waves (averaged over 10 cardiac cycles).

### 2.3. Statistical Analysis

All statistical analyses were performed using Stata® 13 (StataCorp LP, Texas, USA). Baseline characteristics of the study population were evaluated using descriptive statistics. Data were expressed as frequencies and percentages for categorical variables, means ± SD for parametric continuous variables, and median (IQR) for nonparametric continuous variables. The associations between cfPWV and patients' clinical and biological variables were tested using Spearman test for means comparison between 2 groups and Kruskal-Wallis test for associations with categorical variables. To evaluate differences in cfPWV between various etiologies of ESRD, one-way ANOVA with post hoc Bonferroni method of pairwise comparison was performed. Variables with a *p* value ≤ 0.10 in the univariate analysis were held for inclusion in the multivariate regression model, in addition to parameters of interest, and those with historical association with cfPWV. Testing for multicollinearity was performed, and it was predetermined that when variables were strongly correlated with each other, only the variable with the stronger correlation with cfPWV would be retained in the multivariate regression model. All tests were double-sided. A two-sided *p* value ≤ 0.05 was considered statistically significant.

## 3. Results

### 3.1. Patient Characteristics

Patient characteristics are summarized in Table 1. Mean patient age was 52.8 ± 18.5 years, 56% of patients were males, 70% had hypertension, and 23% had diabetes mellitus. The etiology of ESRD was identified among 78% (*n* = 64) of patients and was distributed as follows: vascular renal disease (18.3%, *n* = 15), diabetic nephropathy (18.3%, *n* = 15), nondiabetic glomerulopathy (11%; *n* = 9), tubulointerstitial disease (11%; *n* = 9), and hereditary nephropathy (19.4%; *n* = 16). Among those with vascular renal disease, nephroangiosclerosis was the main etiology of ESRD among 13/15 patients (87%), whereas only 2/15 (13%) were diagnosed with renal artery stenosis. The etiology could not be confirmed among 18 patients due to the lack of identifying clinical features and/or renal biopsy.

### 3.2. Association between cfPWV and Etiology of ESRD

**Figure 1:**
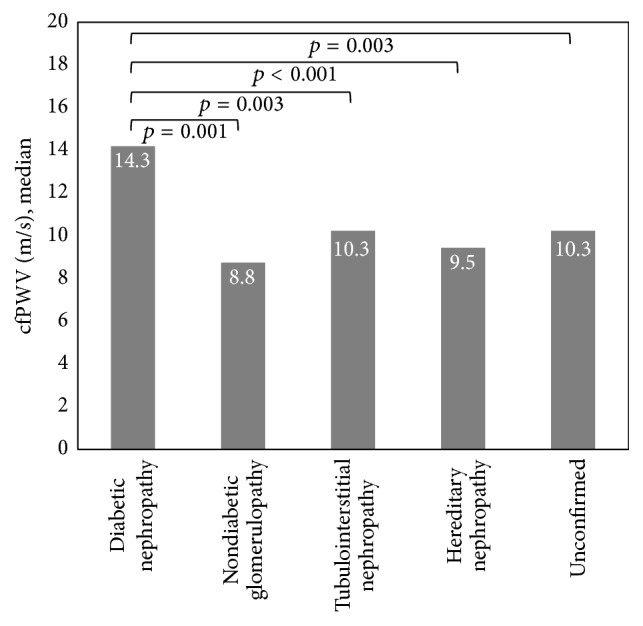
Median cfPWV among patients with diabetic nephropathy as compared with median cfPWV among patients with nondiabetic, nonvascular renal disease.

**Figure 2:**
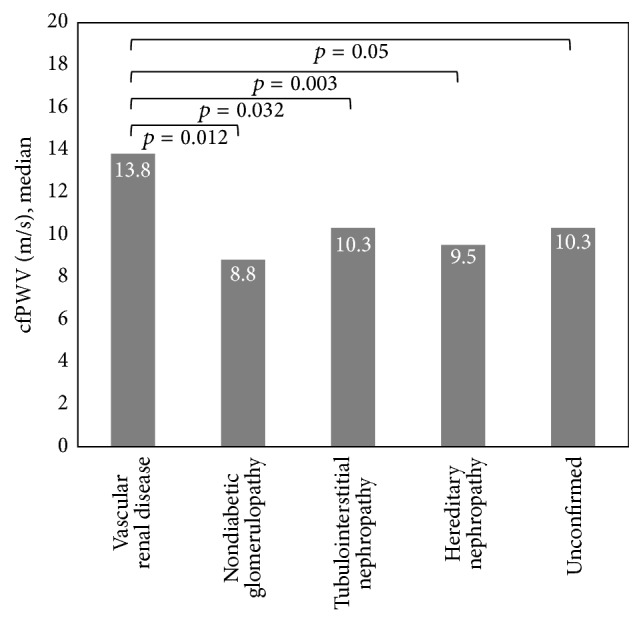
Median cfPWV among patients with vascular renal disease as compared with median cfPWV among patients with nondiabetic, nonvascular renal disease.

**Figure 3:**
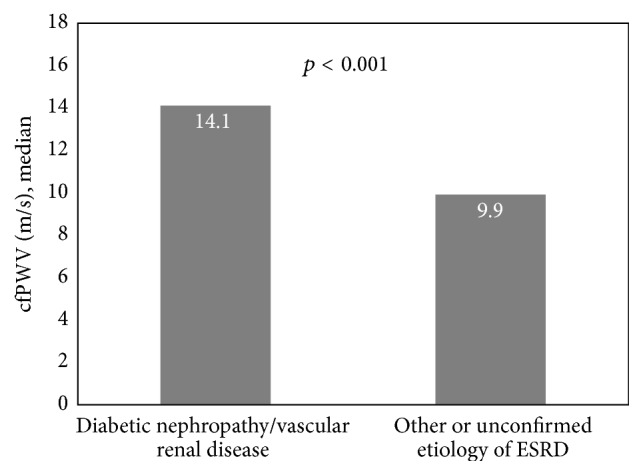
Median cfPWV among patients with either diabetic nephropathy or vascular renal disease as compared with median cfPWV among patients with nondiabetic, nonvascular renal disease.

Univariate analysis demonstrated a significant association between cfPWV and patient age, etiology of ESRD, age at dialysis initiation, length of time on dialysis, weekly dialysis hours, history of hypertension, history of diabetes mellitus, history of hypercholesterolemia, history of CV events, systolic blood pressure, pulse pressure, and iPTH concentration. Multivariate regression adjusted for patient age, length of time on dialysis, weekly dialysis hours, history of hypercholesterolemia, and serum iPTH concentration demonstrated a significant association between cfPWV and vascular renal disease (*p* = 0.036), pulse pressure (*p* = 0.001), and history of CV events (*p* = 0.025), but not diabetic nephropathy (*p* = 0.084), history of hypertension (*p* = 0.58), or history of diabetes mellitus (*p* = 0.74). One-way ANOVA with post hoc Bonferroni method of pairwise comparison demonstrated that median cfPWVs were comparable between patients with diabetic nephropathy (14.3 m/s) and vascular renal disease (13.8 m/s) (Figures 1 and 2), both of which were significantly higher than the median cfPWVs observed among other etiologies of ESRD (9.9 m/s) (*p* < 0.001) (Figure 3). Accordingly, the etiologies of ESRD were then dichotomized into diabetic nephropathy and vascular renal disease versus all other etiologies. Multivariate regression demonstrated a significant, positive association between cfPWV and the composite of either diabetic or vascular nephropathy (*p* = 0.02), when adjusted for all other parameters previously described. Similarly, neither history of hypertension, history or diabetes mellitus, nor the composite of both was significantly associated with cfPWV (Tables 2 and 3).

## 4. Discussion

The impact of increased arterial stiffness on all-cause and CV-related mortality among HD patients has been previously described, and it suggests that the strongest correlates of CV mortality among these patients relate to large artery structure and function [2, 9–12]. The present study provides primary data on the association between the etiology of ESRD and cfPWV among HD patients, where vascular renal disease and diabetic nephropathy were independently associated with higher cfPWV as compared with other etiologies of ESRD. Prior studies have demonstrated that a history of either hypertension or diabetes mellitus is associated with increased arterial stiffness among HD patients [13, 14], but investigations that address the association between the renal sequelae of both diseases and arterial stiffness in this population are scarce [15, 16]. The current results suggest that development of renal disease due to either chronic hypertension (vascular renal disease) or diabetes (diabetic nephropathy) is more strongly associated with arterial stiffness than either disease alone, demonstrating the direct role of the kidneys on arterial stiffness that extends beyond the risks attributed to hypertension and diabetes alone.

Accordingly, these findings reflect the impact of the kidneys on the structure and function of large arteries and the bidirectional relationship between the renal and the vascular systems. More importantly, results from this analysis may help confirm the association between arterial stiffness and CV risk factors and additionally be hypothesis-generating to suggest that more in the clinical context, a more aggressive management may be indicated among ESRD patients due to diabetic or vascular nephropathy as compared with either ESRD patients due to nondiabetic, nonhypertensive etiology or patients with diabetes and hypertension alone.

The role of managing modifiable CV risk factors after HD is not as well understood as it is among patients with CKD without ESRD. Unfortunately, it has been speculated that once patients are on HD, further control of diabetes and blood pressure becomes less pressing, and patient compliance to further control diabetes and blood pressure decreases following the onset of HD. However, this study provides early hypothesis-generating insight to both healthcare professionals and ESRD patients that continuing aggressive control of modifiable CV risk factors is independently associated with improved arterial stiffness and possibly reduced risk of CV events after the onset of HD. Interestingly, the study demonstrated that arterial stiffness among patients who develop ESRD due to modifiable etiologies, such as diabetes and hypertension, is significantly higher than among those with hereditary, nonmodifiable causes. The study suggests that further research is needed to evaluate the role of healthcare professionals in closely monitoring and supporting patients on HD following the onset of HD, and stratification of patients with regard to risk of CV outcomes remains possible after the development of ESRD.

Consistent with prior studies, both pulse pressure and a history of CV events were also associated with cfPWV [8, 17, 18]. Pulse pressure is a result of the intermittent ventricular ejection of blood, which is minimized by the inherent elasticity of large conduit arteries, such as the aorta. This physiological mechanism supports the positive correlation between pulse pressure and arterial stiffness, where stiffer large arteries have a diminished capacity to reduce vascular pulsatility resulting in an increase in pulse pressure [18]. With stiffer arteries, the risk of future CV events increases significantly, and population-based studies have previously demonstrated that an elevated cfPWV is independently associated with an increased CV risk. While a history of CV events is a well-established predictor of future CV events, the current findings confirm the association between a prior history of CV events and arterial stiffness, another surrogate of future CV risk.

In this study, 18 HD patients had ESRD with unconfirmed etiology. Among these patients, there were no clinical indices of either a diagnosis of diabetes mellitus or a history of long-standing hypertension to suggest a diabetic or vascular etiology of ESRD. In addition, the mean age of patients with unconfirmed ESRD etiology was also significantly lower than those with either vascular renal disease or diabetic nephropathy (67.5 years versus 47 years, *p* < 0.001) but was similar to those with ESRD due to either nondiabetic nephropathy, tubulointerstitial nephropathy, or hereditary nephropathy. Accordingly, given their clinical profiles, the etiologies of ESRD in this group of patients were most likely nondiabetic and nonvascular in origin. Compared with cfPWV of patients with vascular renal disease and diabetic nephropathy, cfPWV among patients with unconfirmed renal disease was significantly lower when these patients were analyzed alone and when they were combined with other nondiabetic, nonvascular ESRD etiologies.

The cross-sectional nature of the data should not be considered sufficient to support any recommendation on CKD management based on ESRD etiology or to shed light on the effect of aggressive CV risk factors management on arterial stiffness in HD patients. Results from the current investigation may be considered hypothesis-generating for future research. Although the study is limited by its cross-sectional design, small sample size, monocentric model, and its missing data, the limitations are not thought to significantly affect the validity of the study results. Given the findings from the additional analysis among the 18 patients without a confirmed etiology of ESRD, the interpretation of the missing data does not seem to necessarily alter the interpretation of the study results.

In conclusion, the study suggests that the etiology of ESRD is independently associated with arterial stiffness among hemodialysis patients, and patients with either diabetic nephropathy or vascular renal disease have significantly higher cfPWV than those with other etiologies of ESRD. Furthermore, arterial stiffness was higher among patients who developed renal sequelae of either diabetes mellitus or hypertension as compared with those who have a history of diabetes mellitus or hypertension alone.

## Figures and Tables

**Table 1:** Baseline characteristics of the study population.

Characteristic	Value (*N* = 82)
Age (years), mean **± **SD	52.8 ± 18.5
Male gender, % (*n*)	55% (45)
Age at dialysis initiation (years), mean **± **SD	47.8 ± 19.2
Length of time on dialysis (months), median (IQR)	49.4 (23.7, 87.5)
Consanguinity, % (*n*)	34% (28)
Etiology of ESRD	
Vascular renal disease, % (*n*)	18.3% (15)
Diabetic nephropathy, % (*n*)	18.3% (15)
Nondiabetic glomerulopathy, % (*n*)	11.0% (9)
Tubulointerstitial disease, % (*n*)	11.0% (9)
Hereditary nephropathy, % (*n*)	19.4 (16)
Unconfirmed etiology of ESRD, % (*n*)	22.0% (18)
History of HTN, % (*n*)	71.0 (58)
Pulse pressure (mmHg), mean **± **SD	52.4 ± 14.5
Mean blood pressure (mmHg), mean **± **SD	83.7 ± 12.4
History of diabetes mellitus, % (*n*)	23.2% (19)
History of CV events, % (*n*)	34.2% (28)
History of coronary artery disease, % (*n*)	34.2% (28)
History of stroke, % (*n*)	2.4% (2)
History of significant PAD, % (*n*)	6.1% (5)
Hypercholesterolemia, % (*n*)	52.4% (43)
Active smoking, % (*n*)	34.2% (28)
Calcium (mg/dL), mean **± **SD	8.52 ± 0.92
Phosphorus (mg/dL), mean **± **SD	5.20 ± 1.86
iPTH (ng/L), mean **± **SD	494.7 ± 378.3

CV: cardiovascular; ESRD: end-stage renal disease; HTN: hypertension; iPTH: intact parathyroid hormone level; IQR: interquartile range; PAD: peripheral artery disease; SD: standard deviation.

**Table 2:** Association between cfPWV and the composite of either diabetic nephropathy or vascular renal disease.

Variable	cfPWV (m/s) *R*^2^ = 0.55, *p* ≤ 0.001		
	Coefficient	95% CI	*p* value
Composite of either diabetic nephropathy or vascular renal disease	2.4	0.4, 4.4	0.022
Pulse pressure	0.1	0.03, 0.1	0.001
History of CV events	1.7	0.2, 3.2	0.025
History of hypertension	0.3	−0.9, 1.6	0.59
History of diabetes mellitus	−0.3	−3.1, 2.6	0.84

Multivariate model adjusted for age, length of time on dialysis, weekly dialysis hours, history of hypercholesterolemia, and serum iPTH concentration.

CI: confidence interval; CV: cardiovascular; cfPWV: carotid femoral pulse wave velocity.

**Table 3:** Association between cfPWV and either diabetic nephropathy alone or vascular renal disease alone.

Variable	cfPWV (m/s) *R*^2^ = 0.55, *p* ≤ 0.001		
	Coefficient	95% CI	*p* value
Vascular renal disease	2.3	0.2, 4.5	0.036
Diabetic nephropathy	2.5	−0.4, 5.4	0.084
Pulse pressure	0.08	0.03, 0.1	0.001
History of CV events	1.7	0.2, 3.2	0.025
History of hypertension	0.4	−0.9, 1.6	0.58
History of diabetes mellitus	−0.4	−2.9, 2.1	0.74

Multivariate model adjusted for age, duration of dialysis, hours of dialysis per week, history of hypercholesterolemia, and serum iPTH concentration.

CI: confidence interval; CV: cardiovascular; cfPWV: carotid femoral pulse wave velocity.
